# Deep Learning-Based Artificial Neural Network-Cellular Automata Model in Constructing Landscape Gene in Shaanxi Ancient Towns under Rural Revitalization

**DOI:** 10.1155/2022/1340038

**Published:** 2022-07-16

**Authors:** Xiameng Wei

**Affiliations:** School of Design and Art, Xijing University, Xi'an City 710000, China

## Abstract

With the development of modern industrialization, the rational planning of land resources, especially rural settlements (RSs), has become an important part of rural revitalization. Optimizing the RS spatial layout and enhancing its evolution simulation can encourage effective land resource allocation. It helps improve rural residents' production and living standards, alleviates the pressure of urban and rural land conflicts, and promotes the common development of urban and rural economies. This work mainly uses the ANN-CN (artificial neural network-cellular automata) model to study the cultural landscape gene construction of ancient towns in Shaanxi. First, RSs' spatial layout and landscape pattern index (LPI) in Shaanxi are analyzed. Second, the ANN-CA model is designed using the artificial neural network (ANN). Finally, Hua Yang Ancient Town in southern Shaanxi, Feng Huo Town in Guan Zhong, and Zhong Jiao Town in Northern Shaanxi are selected as the research objects. The influencing factors of spatial layout are extracted, and the evolution extracted of spatial layout is simulated based on the ANN-CA model. The simulation experiment finds that the area change of Hua Yang Ancient Town is more obvious than that of the other two ancient towns, with a change rate of 6.98%. Although the area change rate of Feng Huo Town is 19.79%, the actual change area is less than that of Hua Yang Ancient Town. Second, comparing the simulation accuracy of the model under different parameters, we can obtain the most suitable parameter for predicting the ancient towns' land-use type and construing landscape gene land. Specifically, for Hua Yang Ancient Town, *T* = 0.9 and the random disturbance parameter *α* = 1.0. For Zhong Jiao Town, *T* = 0.8 and *α* = 1.0. For Feng Huo Town, *T* = 0.9 and *α* = 1.0. It is hoped that this work can further carry forward Shaanxi's traditional culture and carry out the protective development of traditional rural buildings.

## 1. Introduction

With China's rapid economic development, urbanization also expands in scale and depth. As a result, many first-tier cities swallow up the coterminous rural lands, thus creating an overcrowded urban population. This trend is still accelerating with more people living in cities than in the countryside [1]. Thanks to the proposal of the even and harmonious urban and rural development policies, rural areas have also seen tremendous growth. The rural settlements (RSs) replanning and development are essential in rural land construction and transformation. At present, the traditional down and dirty RSs can no longer adapt to the rapid social development. The rural public infrastructure cannot meet the residents' increasingly higher material and spiritual pursuits [2]. Therefore, the research on the spatial layout of RSs is of great significance for coordinating urban and rural development and improving residents' production and living conditions. With the deepening construction of new socialist countryside, the contradiction between the limited quantity of land resources and the growth of social demand has become increasingly acute, riveting regional land use's immediate attention [3]. Thus, promoting the rational layout of RSs, urbanization, and the production and living conditions has become urgent for new rural construction. Scientific planning of RSs with local features, rational land allocation, and urban-rural integration are now catching the eyes of many scholars and government authorities. At present, China's rural land structure has some prominent issues, such as large-scale residential land, low efficiency, low land utilization, idle residential land, and waste of cultivated land resources. Such situations are not conducive to sustainability and coordination between land resource utilization and augmented residential needs [4].

Kim and Kim proposed a collaborative modeling method based on big data, machine learning (ML), and simulation modeling. Specifically, the hypothetical model was constructed by cellular automata (CA). The parameters and functions of the hypothetical model were simulated by applying an artificial neural network (ANN). The numerical results showed that the proposed method could apply to the traffic model to predict rural traffic congestion in an unsteady state [5]. Mishra et al. constructed a new clustering analysis method, which inherited the best characteristics of functional link ANN and self-organizing feature mapping. The proposed method employed Chebyshev, Legendre orthogonal polynomial, and power series to map the input data to a new high-dimensional feature space. Then, the objects were clustered according to the competitive learning principle of self-organizing feature mapping. The algorithm's effectiveness was tested on various artificial and real datasets, including remote sensing images. Compared with other common clustering algorithms, the proposed method had a good application prospect in clustering rural datasets [6]. Li et al. applied deep learning (DL) technology to geographic image recognition. Consequently, a multimode image method fused with DL was proposed. The proposed method could significantly improve the fusion effect, image detail definition, and time efficiency [7]. The research results on the spatial layout of RSs are abundant, and many scholars have conducted in-depth research. Based on the research progress and application perspective in China and overseas, there are still some deficiencies: (1) the existing studies mainly focus on the qualitative analysis of socioeconomic factors and the quantitative analysis of a few natural conditions, lacking the quantitative research combining the two. (2) The research on the spatial layout of RSs tends to focus on a large area, lacking reasonable zoning and target planning. More attempts are made to solve a series of regional RS layout optimization problems with unified standards and methods, lacking pertinence. This work mainly studies how to reconstruct the Cultural Heritage Landscape (short for landscape) Gene of ancient towns in Shaanxi to promote the development of traditional culture in Shaanxi. First, the spatial layout of RSs in ancient towns in Shaanxi is understood. Second, the ancient towns' landscape pattern index (LPI) is introduced. Then, the ANN-CNN model is proposed using neural network technologies to predict the landscape's future construction in the ancient towns of Shaanxi. The innovation of the research scheme is to build a prediction model (ANN-CA) using neural network technologies and cellular automata. Afterward, the optimal model parameter with the best accuracy is determined through simulation experiments on the spatial layout of RSs in Hua Yang Ancient Town in southern Shaanxi, Feng Huo Town in Guan Zhong, and Zhong Jiao Town in northern Shaanxi, respectively. Finally, the simulation results are optimized. It is hoped that this study can help landscape in Shaanxi ancient towns develop better and protect and carry forward Shaanxi ancient buildings.

First, the research background and significance are explained. It summarizes the research status of the spatial layout of RS and the simulation of land-use evolution in China and overseas and discusses its shortcomings. The main research content and technical route are put forward on this basis. Furthermore, it focuses on the concept and changes in the spatial layout of RSs. The main concepts of LPI are introduced, and the calculation methods of each LPI are listed in detail. The cellular automata (CA) model and artificial neural network (ANN) model are described in detail by explaining their advantages in spatial layout simulation. Finally, it designs and implements the spatial layout evolution model of RSs.

## 2. Research Scheme Design

### 2.1. Spatial Layout of Rural Settlements (RSs)

The evolution of the spatial layout of RSs has experienced a simple-to-complex and a disorder-to-order process. This evolution is driven by the joint restriction and influence of the internal and external environment, as detailed below.

#### 2.1.1. Socioeconomic Development

RSs were initially built based on natural conditions, where residents were primarily engaged in agricultural production. With the deepening economic reform, RSs no longer rely solely on natural conditions. Nowadays, RSs see more diverse development patterns involving the first, second, and tertiary industries. The reconstruction and upgrading of the industrial structure have revolutionized the spatial layout and drove RSs towards large-scale and intensive development [8].

#### 2.1.2. Production and Living Needs

Based on economic development, the quality of production and life of residents has been improved and so is the living environment of RSs. The traditional rural infrastructure is poor and unable to meet the residents' basic needs, such as schools and medical treatment. Over time, the Chinese RSs have gradually evolved to places with excellent infrastructure. Additionally, the traditional rural transportation conditions are poor, hindering production and life [9].

#### 2.1.3. Government Policy Driven

In order to implement the National Rural Revitalization Strategy and further optimize the spatial layout of villages and towns, the government will also guide the classified improvement of the living environment of RSs. The overall socioeconomic advancement impels rural economic development. The upgrading and transformation of rural industries are indispensable from the state macrocontrol. Only government can guide the practical evolution of RSs' spatial layout from disorderly to orderly [10].

### 2.2. Landscape Pattern Index (LPI)

LPI refers to landscape pattern and landscape index. It is a simple quantitative index that condenses landscape patterns and reflects that LPI can establish the relationship between landscape and pattern process. Thus, it is often used to quantitatively express the differences and changes in the spatial pattern [11].

RS landscapes are a mosaic composed of natural and cultural patches of different sizes, shapes, and combinations. The LPI of landscape ecology can quantitatively analyze the distribution of different landscape patterns in typical cities and towns, making the spatial pattern analysis more intuitive and scientific [12]. Here, the patch number, average patch area, maximum patch index, patch density, minimum adjacent distance, patch shape index, and patch fractal dimension index are selected to analyze the landscape pattern characteristics of RSs in Shaanxi Province.

Number of patches (NP):

(1) $$\begin{array}{c} NP=N, \end{array}$$

where *N* represents the patch number of landscape elements of RSs [13].

Average patch area (AREA_MN):

(2) $$\begin{array}{c} \text{AREA}\_\text{MN}=\frac{1}{N}\sum_{j=1}^{N_{i}}A_{j}, \end{array}$$

where *A*_*j*_ represents the area of the *j*-th patch of RS landscape elements.

Largest patch index (LPI):

(3) $$\begin{array}{c} \text{LPI}=\frac{n}{A}\times 100\%, \end{array}$$

where *n* represents the largest patch area of RS landscape elements.

Patch density (PD):

(4) $$\begin{array}{c} \text{PD}=\frac{N}{A}\times 100\%, \end{array}$$

where *A* represents the total area of landscape elements of RSs.

Minimum adjacency distance (ENN_MN):

(5) $$\begin{array}{c} \text{ENN}\_\text{MN}=\text{MIN}\left(D\right), \end{array}$$

where *D* represents the distance between RS landscape elements.

Patch shape index (SHAPE_MN):

(6) $$\begin{array}{c} \text{SHAPE}\_\text{MN}=\frac{0.25P}{\sqrt{A}}, \end{array}$$

where *P* indicates the patch perimeter of RS landscape elements.

Patch fractal dimension (FRAC_MN):

(7) $$\begin{array}{c} \text{FRAC}\_\text{MN}=\frac{2\text{ln}\left(P/k\right)}{\ln\left(A\right)}, \end{array}$$

where *k* is a constant with a value of 4.

In equation (7), NP refers to the number of landscape elements of RSs. AREA_MN means the ratio of the total patch area of all RSs to the number of patches. The larger the AREA_MN is, the larger the scale of RSs is [14]. LPI denotes the ratio of the largest patch area to the total area of RSs. The larger the LPI is, the larger the patch size of RSs is, and vice versa. PD is RSs/unit area. ENN_MN refers to the minimum RS elements distance. SHAPE_MN represents the complexity of the patch. Lastly, FRAC_MN stands for the degree of irregularity of RSs [15].

### 2.3. Artificial Neural Network (ANN)

ANN features such functions as self-learning, self-adaptation, self-organization, and robust nonlinear mapping. It is especially suitable for uncertain reasoning, judgment, identification, and classification of causality [16]. The core function of ANN is “training.” A batch of predefined input/output data is used to analyze and summarize the potential laws between them. The new rules are combined with the input data to predict the output. Like the biological nervous system, the artificial neuron is the basic unit of ANN and the basic mathematical model for simulating biological neurons [17, 18]. As a nondynamic nonlinear function, the output function of the neuron model is usually used to simulate the nonlinear characteristics of nerve cells, such as excitation, inhibition, and threshold.

Each input connection point of the neuron uses the connection weight to indicate the apparent connection's strength and amplifies the upcoming signal's strength during the connection. The state variables of each node have corresponding connection weight coefficients. The processing unit quantizes the weighted input and sums the weighted values to calculate the individual output [19]. So far, many kinds of ANNs have been developed. Among them, the multilayer feedforward network is widely used, which is composed of the input, output, and hidden layers. The present work selects the backpropagation neural network (BPNN) model to integrate with CA. Then, the system errors are collected and returned to the simulation process to adjust the neuron weight [6, 20].

**Figure 1:**
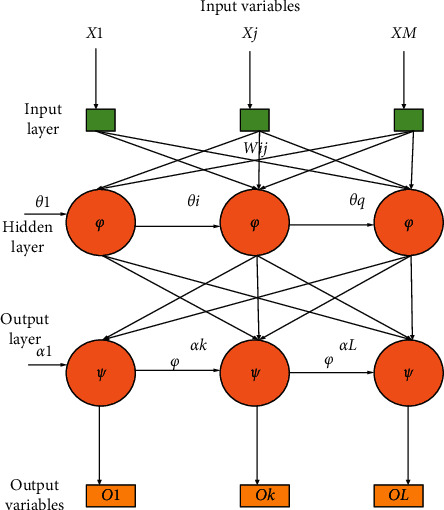
Structure of BPNN.

BPNN is a multilayer feedforward neural network (FNN) characterized by forwarding signal propagation and error backpropagation. Generally, the operation process of the BPNN is divided into two stages. The first stage is the forward signal propagation of the input layer->hidden layer->output layer. The second stage is the error backpropagation of the output layer->hidden layer->input layer. The weights and biases from the input layer to the hidden and output layers are adjusted in turn [21].  Figure 1 shows the structure of the BPNN.

The *j*-th inputted node in the input layer is denoted as *x*_*j*_, and the value range of *J* is [1, *M*].

In the hidden layer, the weight from the *i-*th node to the *j*-th node of the output layer is generally represented by *w*_*ij*_.

In the hidden layer, *θ*_*i*_ represents the threshold of the node [22].

The activation function (AF) in the hidden layer is generally expressed by Φ(*x*).

*w*_*ki*_ represents the weight between the *k*-th node of the output layer and the *i*-th node of the hidden layer, where *i* = 1,…,q.

In the output layer, *α*_*k*_ represents the value of node *K* (*K*∈ [1, l]).

The AF is generally represented in the output layer by *ψ*(*x*) [23].

In the output layer, *O*_*k*_ represents the node output.

First, the signal forward propagation process is as follows.

The input net_*i*_ of the *i*-th node of the hidden layer is calculated by using the following equation:

(8) $$\begin{array}{c} {\text{net}}_{i}=\sum_{j=1}^{M}w_{ij}x_{j}+\theta_{i}. \end{array}$$

Output *y*_*i*_ of the *i*-th node of the hidden layer is counted by using the following equation:

(9) $$\begin{array}{c} y_{i}=\Phi \left({\text{net}}_{i}\right)\\ =\Phi \left(\sum_{j=1}^{M}w_{ij}x_{j}+\theta_{i}\right). \end{array}$$

The calculation of input net_*k*_ of the *k*-th node of the output layer reads

(10) $$\begin{array}{c} {\text{net}}_{k}=\sum_{i=1}^{q}w_{ij}x_{j}+a_{k}\\ =\sum_{i=1}^{q}w_{ij}\Phi \left(\sum_{j=1}^{M}w_{ij}x_{j}+\theta_{i}\right)+\alpha_{k}. \end{array}$$

Output *O*_*k*_ of the *k*-th node of the output layer is expressed by the following equation:

(11) $$\begin{array}{c} O_{k}=\psi \left({\text{net}}_{k}\right)\\ =\psi \left(\sum_{j=1}^{M}w_{ij}x_{j}+\theta_{k}\right)\\ =\psi \left(\sum_{i=1}^{q}w_{ki}\Phi \left(\sum_{j=1}^{M}w_{ij}x_{j}+\theta_{i}\right)+\alpha_{k}\right). \end{array}$$

Second, the error backpropagation process is as follows.

From the output, error backpropagation calculates the error of neurons layer by layer. Then, the weight and threshold of each layer are adjusted according to the error gradient descent method (GDM) to get an output as close to the expected value as possible [24–26].

For each sample *p*, the quadratic error criterion function *E*_*p*_ is calculated by the following equation:

(12) $$\begin{array}{c} E_{p}=\frac{1}{2}\sum_{K=1}^{L}{\left(T_{k}-O_{k}\right)}^{2}. \end{array}$$

The total error criterion function of the system for *P* training samples is counted by the following equation:

(13) $$\begin{array}{c} E=\frac{1}{2}\sum_{P=1}^{P}\sum_{K=1}^{L}{\left(T_{k}^{p}-O_{k}^{p}\right)}^{2}. \end{array}$$

Then, the correction amount △*w*_*ki*_ of output layer weight, output layer threshold △*a*_*k*_, hidden layer weight △*w*_*ij*_, and hidden layer threshold △*θ*_*i*_ are corrected in turn according to the error GDM.

(14) $$\begin{array}{c} △w_{ki}=-\eta \frac{\delta E}{\delta w_{ki}}, \end{array}$$

(15) $$\begin{array}{c} △a_{k}=-\eta \frac{\delta E}{\delta a_{k}}, \end{array}$$

(16) $$\begin{array}{c} △w_{ij}=-\eta \frac{\delta E}{\delta w_{ij}}, \end{array}$$

(17) $$\begin{array}{c} △\theta_{i}=-\eta \frac{\delta E}{\delta \theta_{i}}. \end{array}$$

The output layer's weight adjustment equation reads

(18) $$\begin{array}{c} △w_{ki}=-\eta \frac{\delta E}{\delta w_{ki}}\\ =-\eta \frac{\delta E}{\delta O_{K}}\frac{\delta O_{K}}{\delta {\text{net}}_{k}}\frac{\delta {\text{net}}_{k}}{\delta w_{ki}}. \end{array}$$

The output layer's threshold adjustment equation reads

(19) $$\begin{array}{c} △a_{k}=-\eta \frac{\delta E}{\delta a_{ki}}\\ =-\eta \frac{\delta E}{\delta O_{K}}\frac{\delta O_{K}}{\delta {\text{net}}_{k}}\frac{\delta {\text{net}}_{k}}{\delta w_{k}}. \end{array}$$

The weight adjustment equation of the hidden layer reads

(20) $$\begin{array}{c} △w_{ij}=-\eta \frac{\delta E}{\delta w_{ij}}\\ =-\eta \frac{\delta E}{\delta O_{i}}\frac{\delta O_{i}}{\delta {\text{net}}_{i}}\frac{\delta {\text{net}}_{i}}{\delta w_{ij}}. \end{array}$$

The hidden layer's threshold adjustment equation reads

(21) $$\begin{array}{c} △\theta_{i}=-\eta \frac{\delta E}{\delta \theta_{i}}\\ =-\eta \frac{\delta E}{\delta y_{i}}\frac{\delta y_{i}}{\delta {\text{net}}_{i}}\frac{\delta {\text{net}}_{i}}{\delta \theta_{i}}. \end{array}$$

In equations (18)–(21), *w*_*ki*_ is the output layer weight, *a*_*ki*_ denotes the output layer threshold, *w*_*ij*_ indicates the hidden layer weight, *θ*_*i*_ represents the hidden layer threshold, net refers to the node, and *O*_*i*_ signifies the node output.

## 3. Landscape Gene Extraction and Identification

This section selects Shaanxi ancient towns for landscape gene extraction and identification [27].

**Figure 2:**
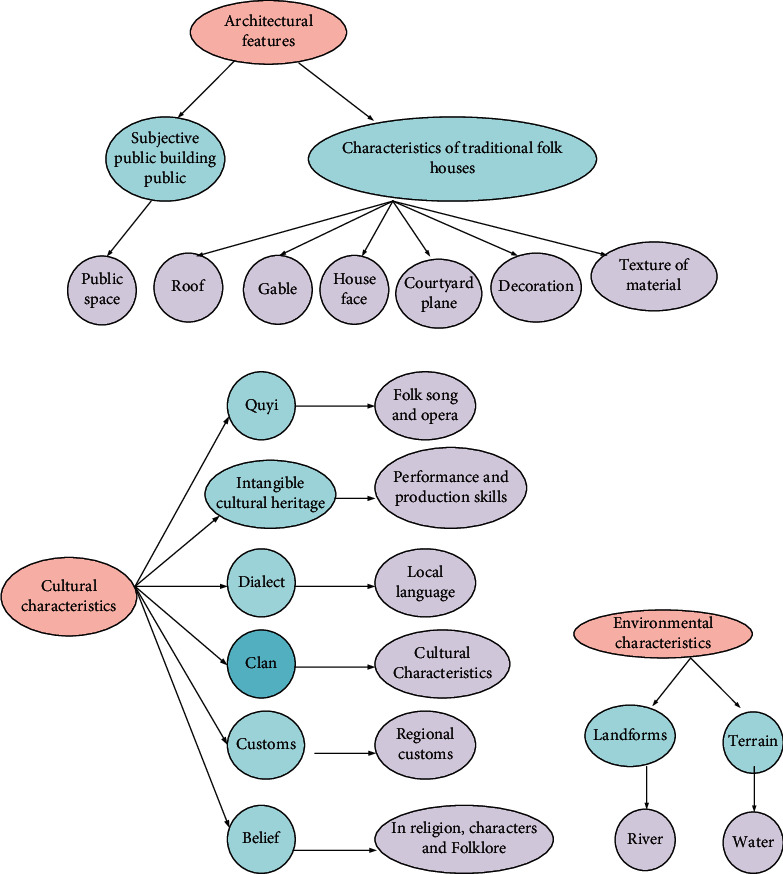
Shaanxi traditional RSs' landscape gene-oriented FEI index system: (a) architectural feature extraction; (b) cultural feature extraction; (c) environmental feature extraction.

Figure 2 gives the landscape gene-oriented feature extraction and identification (FEI) index system in traditional Shaanxi RSs.

The connections in Figure 2 are the process of extracting the morphological characteristics of the ancient town landscape. Based on this, the cultural environment characteristics and landscape gene characteristics of the ancient town landscape are analyzed from the macro-, meso-, and microscales. Then, the regional cultural characteristics of the ancient town are identified to better understand and protect the ancient town's landscape from the perspective of cultural geography and provide a scientific basis for the cultural revival of ancient towns.

According to the landscape gene theory, landscape gene extraction and identification mainly include landscape gene resource management and recognition operations. According to the unique characteristics of Shaanxi traditional RS landscape, the landscape gene identification process is mainly divided into the following steps:

- Establishment and optimization of the index system: according to the unique characteristics of RSs in Shaanxi, the present work proposes a landscape gene-oriented FEI index system for the traditional RSs in Shaanxi [28].
- Identification principles and methods: the proposed FEI index system adheres to the principles of “internal uniqueness, external uniqueness, local uniqueness, and overall superiority.” It integrates element extraction, pattern extraction, structure extraction, and meaning extraction. As a result, the proposed FEI index system maintains landscape genes' integrity, authenticity, and accuracy using research and interview [29].
- Deconstruction of the characteristics of traditional RSs and interpretation of historical and cultural context: according to the basic data sorted out and the field investigation, the characteristics of traditional RSs are deconstructed. Then, the historical and cultural context of each RS is sorted out. Finally, each RS's development context and evolution characteristics are grasped from a macroperspective.
- The specific operation of landscape gene extraction and identification: landscape genes are identified and extracted one by one using the proposed landscape gene-oriented FEI index system for Shaanxi traditional RSs.
- Analysis of landscape gene features: the recognition results can be used to analyze the features of the corresponding RS landscape and general laws.
- Then, the gene database of Shaanxi traditional RS landscape is established using the identification results. The database lays a solid foundation for constructing Shaanxi's traditional RS Landscape Genome Map and regional landscape gene FEI system.

### 3.1. Basic Features of Landscape Gene

#### 3.1.1. Southern Shaanxi

Southern Shaanxi, namely, the southern part of Shaanxi Province, is mainly represented by the Han Zhong area. It has been known as “Little Jiangnan in Northwest China” since ancient times. Han Jiang Valley is sandwiched between Qin Ling Mountains and Ba Shan Mountains in Sichuan from north to south. Thus, it is characterized by the terrain of “two mountains and one river.” Mountains, rivers, and gullies are connected, water systems extend in all directions, and natural resources are affluent.

The southern Shaanxi ancient towns were mainly formed after merchants gathered for trade. These settlements are mainly located in the peaceful and flat hillside area or the plain between the mountain valleys. Moreover, many settlements are distributed near the river. Few settlements are grounded on the plain-dominate terrain except the Le Feng Village in Cheng Gu County. Han Zhong is located in the west of southern Shaanxi. Many villages gather along the Qin Ba Ancient Road, such as the old county village and Hua Yang Ancient Town on Tang Luo Ancient Road, Miao Taizi Village, and Cheng Guan Village on Baoxie Ancient Road. They have witnessed the prosperity of the Qin Ba Ancient Road in the past. Therefore, the present work selects Hua Yang Ancient Town as the representative ancient town in southern Shaanxi.

#### 3.1.2. Guan Zhong Region

Historically, the Guan Zhong area has long been the capital of feudal dynasties. The idea of the imperial city influences the people living in Guan Zhong. They have high requirements for the location of settlements. On the other hand, Guan Zhong Plain is vast. In the face of frequent wars, being devoid of geographical advantage, the traditional settlements often prioritize external defense at the construction site. Therefore, most villages in Guan Zhong Plain are constructed as ancient defensive castles based on careful consideration. They have become the main national-level ancient towns selected as Chinese cultural heritage. According to site selection, such villages are mainly divided into two categories. The first type of village uses the Yellow River (the second longest river in China and is believed to be the birthplace of the Chinese civilization) as the natural barrier. They included He Yang and Han Cheng in Wei Nan City. The other category chooses to build high walls with deep moats around the main city areas. At the same time, under the influence of traditional ideas, the city's shape is primarily square castles. Therefore, this section selects Feng Huo Town, Li Quan County, as the representative of ancient towns in the Guan Zhong area.

#### 3.1.3. Northern Shaanxi

Northern Shaanxi is located in the north of Shaanxi, bordering Inner Mongolia. Most residents here settle in caves. Cave dwellings are one traditional Chinese architectural form. It is mainly created by residents of the Loess Plateau (a highland area in north-central China, covering much of Shanxi, northern Henan, Shaanxi, and eastern Gansu provinces, and the middle of the Yellow River). These cultural heritages are deemed one of China's oldest forms of dwellings. Cave settlements are RSs living in caves. At present, cave settlements are still one of the main settlement modes in China, which are widely distributed in Ningxia, Shanxi, Shaanxi, and other places. Accordingly, Zhong Jiao Town of Suide County is selected to represent ancient towns in northern Shaanxi.

### 3.2. Building ANN-CA Model

Traditional linear methods cannot accurately extract the transformation rules of land-use change, and the ANN model has good nonlinear processing ability. Therefore, the ANN-CA model is constructed to study the evolution of rural residential spatial layouts.

**Figure 3:**
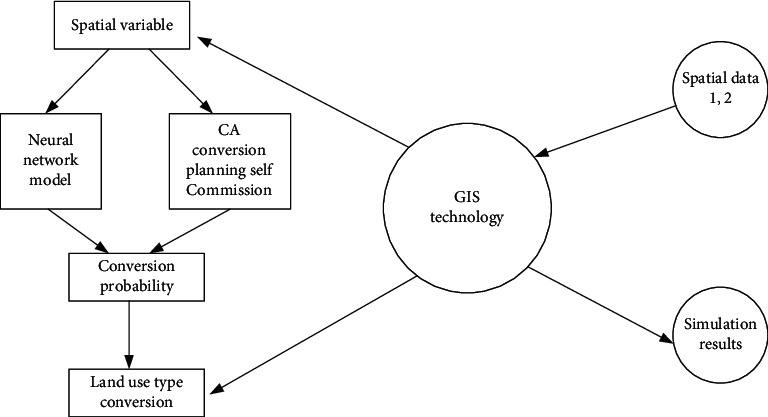
ANN-CA flowchart.

The main operation process of the ANN-CA model is unfolded below. Use GIS (Geographic Information Systems) technology to convert spatial data into training data convenient for model calculation. Take the transformed influencing factors of spatial layout evolution and neighborhood cell data as variables for random sample extraction. Finally, simulate the evolution of land-use types by setting model threshold, training times, random interference items, simulation accuracy, and other conditions.  Figure 3 sketches the flowchart of the ANN-CA model.

In this work, ANN is selected to couple with the CA model. The ANN model includes an input layer, hidden layer, and output layer. Of these, the input layer includes 13 neurons, corresponding to 13 spatial variables, including six land-use types and influencing factors, such as traffic road, elevation, and population density. These variables play a decisive role in the model calculation. Through the calculation of the hidden layer, the conversion probability of land types is output at the output layer. Therefore, the output layer includes six neurons.

The CA model has powerful spatial computing ability and can effectively simulate complex dynamic evolution processes. However, its transformation rules are mainly obtained through the linear method, which is easily affected by subjective factors. The representation method is relatively simple. There are some deficiencies in the simulation of land-use change. Additionally, the evolution of the spatial layout of RSs is the result of the joint action of multiple factors. Defining the transformation rules must consider the impact of the natural environment, geographical location, social economy, and other factors. The traditional linear method cannot accurately extract the conversion rules of land-use change, and the ANN model has good nonlinear processing ability. Therefore, the ANN-CA fusion model is designed to study the evolution of RS spatial layout. The main operation process of the ANN-CA model is as follows. GIS technology converts spatial data into training data convenient for model calculation. The transformed influencing factors of spatial layout evolution and neighborhood cell data are taken as variables for random sample extraction. Then, the evolution of land-use types is simulated by setting the model threshold, training times, random interference items, simulation accuracy, and other conditions. The simulated land-use data of the three cities and towns are compared with the actual land-use data. If the accuracy requirements are met, the trained model is used to predict the land use data of the three cities and towns, respectively. Otherwise, iterative simulation experiments are carried out by constantly modifying the threshold, the number of training samples, and other conditions until the simulation results meet the accuracy requirements.

The model data obtained from data processing include geographic location, topographic, socioeconomic, and adjacent land-type data. The geographical location variables include the distance from the road and the distance from the river. The distance from each grid point to the road and the river needs to be calculated by software. Topographic and geomorphic data include elevation and slope, which are obtained by processing elevation data with software. Socioeconomic statistics, including population density and total fiscal revenue, are obtained from the Yearbook. The neighborhood land-use types are mainly six land-use types: agricultural land, RSs, forest land, water body, industrial and mining construction land, and other land use extracted from remote sensing image classification. The vector data of land use are transformed into grid data, which is the basis of model data. As the town area is taken as the research unit in this work, the research area is relatively small. Therefore, the grid data are converted to 20^∗^20 images through resampling. In this way, the grid data can effectively support the model training. The raster data are exported and converted into program-readable.txt files by software.

## 4. Analysis of Experimental Results

### 4.1. Analysis of Land-Use Types in Different Ancient Towns

**Figure 4:**
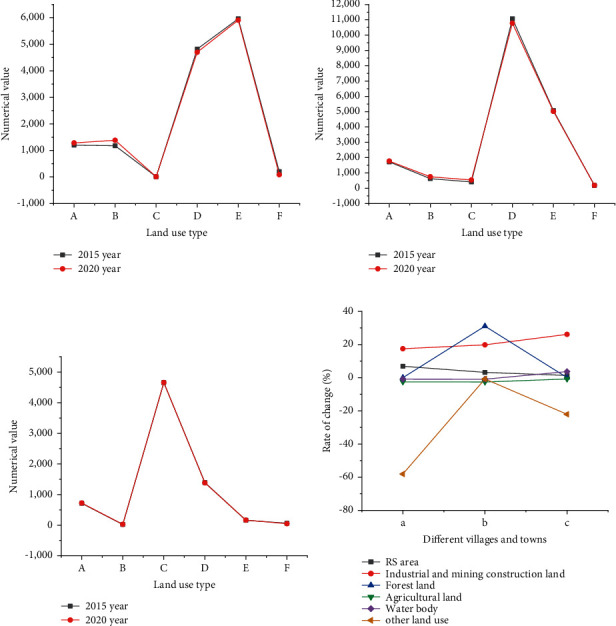
Areas of different land-use types in villages and towns (A: RS area; B: industrial and mining construction land; C: forest land; D: agricultural land; E: water body; F: other land use). (a) Hua Yang ancient town in southern Shaanxi; (b) Feng Huo Town in Guan Zhong area; (c) Zhong Jiao Town in northern Shaanxi; (d) change rate of land-use type.

Figure 4 shows land-use types' investigation and research results in different ancient towns.

Figure 4 compares the land area changes of Hua Yang Ancient Town in southern Shaanxi, Feng Huo Town in Guan Zhong, and Zhong Jiao Town in northern Shaanxi. Apparently, the area change of Hua Yang Ancient Town is more evident than that of the other two ancient towns, with a change rate of 6.98%. Although the area change rate of Feng Huo Town is 19.79%, the actual change area is less than that of Hua Yang Ancient Town. Presumably, the area of Feng Huo Town is relatively small, resulting in a high rate of change. In terms of agricultural land, the land change rate of Zhong Jiao Town is the lowest. The change rates of Hua Yang Ancient Town and Feng Huo Town are 2.51% and 2.62%, respectively. Zhong Jiao Town is probably short of water resources, located in the Loess Plateau. Among other land-use types, the area change of Hua Yang Ancient Town is the most prominent, followed by Zhong Jiao Town. By comparison, Feng Huo Town has almost no land-use change (only 0.77%).

### 4.2. Landscape Index Analysis of Different Ancient Towns

**Figure 5:**
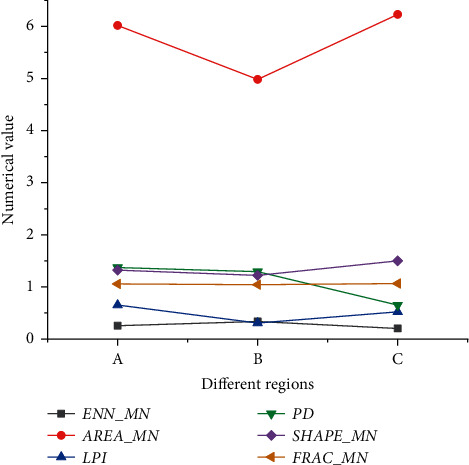
Statistics of landscape index analysis results. (a) Hua Yang Ancient Town in southern Shaanxi; (b) Feng Huo Town in Guan Zhong; (c) Zhong Jiao Town in northern Shaanxi.

Figure 5 plots the numerical statistics of landscape index analysis of three ancient towns.

As in Figure 5, the average patch area (AREA_MN) of Hua Yang Ancient Town and Zhong Jiao Town is 6.02 hectares and 6.23 hectares, and Feng Huo Town in Guan Zhong is the least. Hence, Zhong Jiao Town's land area is more significant than that of Feng Huo Town. The comparison results also suggest that Hua Yang Ancient Town is mainly industrial. The villagers are closely connected. The settlement distribution is dense, and the patch density is the highest. In contrast, Zhong Jiao Town has the lowest patch density. The reason behind it is the small number of villagers who prefer living more intensively, so the minimum adjacent distance is the smallest. Conversely, the minimum adjacent distance of Feng Huo Town is the largest.

### 4.3. Model Accuracy Analysis

**Figure 6:**
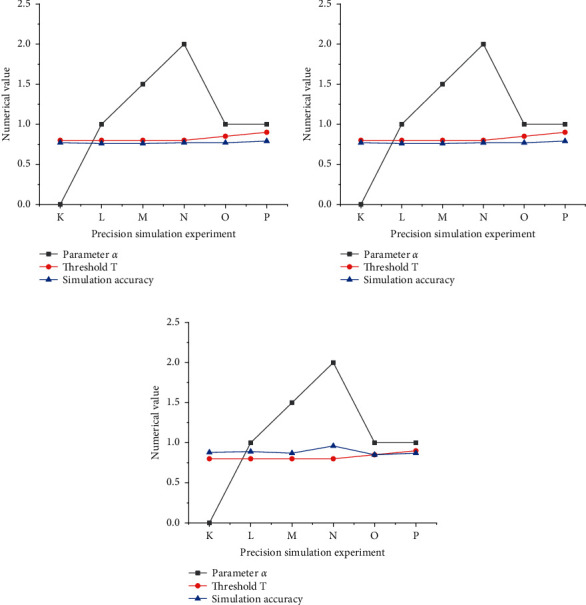
Analysis results of model accuracy on different villages and towns. (a) Hua Yang Ancient Town in southern Shaanxi; (b) Feng Huo Town in Guan Zhong area; (c) Zhong Jiao Town in northern Shaanxi. K-P stands for simulation experiment number.

Figure 6 analyzes the model accuracy for three ancient towns.

Figure 6 compares the simulation accuracy of the proposed ANN-CA model in different villages and towns. First, the model accuracy on Hua Yang Ancient Town in southern Shaanxi is not high (yet within an acceptable range). It is because the land type of Hua Yang Ancient Town changes frequently. Second, by comparing model accuracy under different parameters, the optimal model parameters, threshold *T* = 0.9 and the random disturbance parameter *α* = 1.0, are obtained for predicting Hua Yang Ancient Town's cultural landscape land use in 2023.

Meanwhile, under an increasing random disturbance parameter *α* and a constant threshold *T*, the accuracy of the proposed ANN-CA model decreases in Feng Huo Town in the Guan Zhong area. Then, as the threshold increases under a constant *α*, the proposed model accuracy increases. Overall, the model accuracy falls within a reasonable range. Accordingly, this work determines the optimal model parameters for predicting the landscape gene land construction of Feng Huo Town in Guan Zhong in 2023.

Lastly, the proposed ANN-CA model accuracy on Zhong Jiao Town in northern Shaanxi is higher than on Hua Yang Ancient Town in southern Shaanxi and Feng Huo Town in Guan Zhong. Possibly, Zhong Jiao Town in northern Shaanxi has not changed dramatically in land-use type, so it is less affected by external factors. Thus, the optimal parameters for predicting Zhong Jiao Town's landscape land use in 2023 are determined: threshold *T* = 0.8 and *α* = 1.0.

### 4.4. Model Accuracy Verification

**Figure 7:**
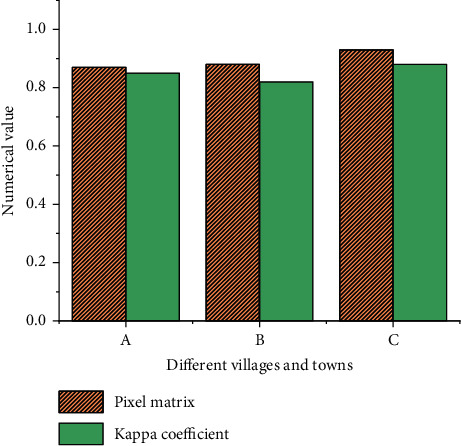
Statistics of prediction accuracy of ANN-CA model for different ancient towns. (A) Hua Yang Ancient Town; (B) Feng Huo Town; (C) Zhong Jiao Town.

Figure 7 collates the prediction accuracy of the proposed ANN-CA model for three ancient towns.

The pixel matrix results of the three ancient towns are close to 1. Hence, the total number of ancient town landscape pixels in the ANN-CA model is close to the total number of actual ancient town landscape pixels. The kappa coefficients of Hua Yang Ancient Town, Feng Huo Town, and Zhong Jiao Town are more than 80%. Thus, the ANN-CA model has high reliability and simulation accuracy. Therefore, overall, the simulation accuracy on Hua Yang Ancient Town, Feng Huo Town, and Zhong Jiao Town meets the prediction requirements.

## 5. Conclusions

This work uses the ANN-CN model to predict and analyze landscape construction in Hua Yang Ancient Town of southern Shaanxi, Feng Huo Town of Guan Zhong, and Zhong Jiao Town of northern Shaanxi. Through the analysis of CA and the cultural model of Shaanxi, the optimal landscape pattern can be obtained. As a result, the ANN-CA model is implemented to realize the optimal landscape design of Shaanxi. The simulation results show that the land change rate of agricultural land in Zhong Jiao Town is the smallest. The change rates of Hua Yang Ancient Town and Feng Huo Town are 2.51% and 2.62%, respectively. Among other land-use types, the area change of Hua Yang Ancient Town is the most obvious, followed by Zhong Jiao Town. By comparison, Feng Huo Town has almost no change (only 0.77%). Second, the kappa coefficients of Hua Yang Ancient Town, Feng Huo Town, and Zhong Jiao Town by the proposed ANN-CA model are greater than 80%. The results prove the model's excellent reliability and accuracy. Because of data limitations, the land-use data selected have a short time interval, which is unstable. At the same time, in terms of socioeconomic conditions, the selected area is small, so there are certain restrictions in the process of searching for the Yearbook. In searching for the factors of socioeconomic conditions, although the Yearbooks of various periods in the region are complete, the Yearbook data are not unified. Thus, few factors are selected which has a certain impact on the simulation of the model. In the follow-up study, the data of a longer period will be used as much as possible and more data will be obtained through the local government. This work involves four categories of influencing factors, but the evolution of the spatial layout of RSs is a very complex process, and it is also a human-oriented evolution process. Human activities also have a certain influence on the evolution of the spatial layout. Therefore, adding residents' subjective will in the follow-up and improving the simulation accuracy through the multiagent model combined with residents' will and government policies are necessary. Meanwhile, the effect of simulation lies in the algorithm. The algorithm selected also has some limitations. It is expected to carry out in-depth research on the conversion rules to make the relevant research more meaningful in the future.

## Data Availability

The data used to support the findings of this study are included within the article.
